# The EOS 3D imaging system reliably measures posterior tibial slope

**DOI:** 10.1186/s13018-021-02529-9

**Published:** 2021-06-16

**Authors:** Andreas Hecker, Till D. Lerch, Rainer J. Egli, Emanuel F. Liechti, Frank M. Klenke

**Affiliations:** 1 Department of Orthopaedic Surgery and Traumatology, Inselspital, Bern University Hospital, University of Bern, Bern, Switzerland; 2 University Institute for Diagnostic, Interventional and Pediatric Radiology, Inselspital, Bern University Hospital, University of Bern, Bern, Switzerland

**Keywords:** Tibial slope, Planning HTO, EOS, Sagittal lower leg alignment, Long leg radiographs

## Abstract

**Background:**

One of the values determined during the assessment of knee issues is the posterior tibial slope (PTS). A new option for measuring the PTS is the EOS 3D imaging system, which provides anteroposterior (AP) and lateral long leg radiographs (LLRs) using less radiation than a conventional LLR. We investigated the reliability of the EOS 3D imaging system with respect to PTS measurements.

**Methods:**

We retrospectively searched our radiological database for patients who underwent an EOS scan and a computed tomography (CT) scan of their lower extremities between January and December 2019. Fifty-six knees were included in the study. Medial and lateral PTSs were determined using both modalities. A radiologist and an orthopaedic surgeon each performed all measurements twice and the intraclass correlation (ICC) was calculated to assess inter- and intrarater reliability. The Student *t* test and Pearson correlation were used to compare the results of both imaging modalities.

**Results:**

The mean medial PTS was 8.5° (95% confidence interval [CI], 8.1–8.9°) for the EOS system and 7.7° (95% CI, 7.3–8.1°) for CT, and the lateral PTS was 7.4° (95% CI, 6.9–7.9°) for the EOS system, and 7.0° (95% CI, 6.5–7.4°) for CT. Interrater reliability (ICC) with respect to medial and lateral PTSs measured on the EOS (0.880, 0.765) and CT (0.884, 0.887) images was excellent. The intrarater reliability of reader 1 (ICC range, 0.889–0.986) and reader 2 (ICC range, 0.868–0.980) with respect to the same measurements was excellent.

**Conclusion:**

The PTS measurements from the EOS 3D imaging system are as reliable and reproducible as those from CT, the current gold standard method. We recommend using this system if possible, because it acquires more information (sagittal plane) in a scan than a conventional LLR, while exposing the patient to less radiation.

**Level of evidence:**

Level III, Retrospective cohort study

## Background

A thorough clinical and radiological assessment of a patient’s knee is mandatory for successful orthopaedic treatment. Many centres are equipped to take anteroposterior (AP) and lateral standard radiographs of the knee as well as AP long leg radiographs (LLRs) for this assessment. One value determined from the radiographs is the posterior tibial slope (PTS). Among other factors, the PTS plays an important role in repeated anterior cruciate ligament (ACL) injuries because an extreme PTS is a known risk factor for rerupturing the ACL and needs to be addressed in re-revision surgery [1]. Moreover, because an extreme PTS can accelerate the development of osteoarthritis (OA) and cause the deterioration of the integrity of the ACL, it should be corrected if a high tibial osteotomy is performed. On the other hand, an increase in the PTS can be beneficial in cases of posterior cruciate ligament (PCL) deficiency [2, 3]. Correct adjustment of the PTS is also a factor in the success of total knee arthroplasty (TKA) [4, 5].

To achieve the desired intraoperative results, a reliable method for measuring the preoperative PTS is crucial. The gold standard is to measure the PTS on computed tomography (CT) images, or at least on lateral full tibia radiographs, but they are not always available [6]. Unfortunately, determination of the PTS on lateral knee radiographs is not precise and leads to overestimation [7, 8]. The tibial axis (TA), defined by the midpoint of the tibial plateau and the midpoint of the ankle joint, is a reliable reference for measuring the PTS, but it is sometimes difficult to determine intraoperatively. The anterior tibial cortex (ATC) is used as an additional reference. In some TKA systems, an intramedullary tibial guide is utilised to determine the TA [9]. Other reference axes, such as the posterior tibial cortex, the fibula axis, or the line connecting the midpoints of the medullary canal at two different heights (circle method) have also been proposed for use in measuring the tibial slope [10, 11].

In 2018, our clinic obtained an EOS 3D imaging system (EOS Imaging, Paris, France), which is now available in many centres worldwide. This system can obtain AP and lateral LLRs as well as full-body radiographs. Two advantages of this system are that it exposes patients to a lower dose of radiation than conventional radiography [12–14] and it uses collimators to generate parallel beams. Conventional radiography uses point-source geometry, which causes spatial distortion [15].

We initially used EOS images to determine frontal plane alignment but then realised that PTS measurements using the lateral plane of the EOS image might be possible and even more accurate than those on lateral knee radiographs. We hypothesised that PTS measurements on EOS radiographs are as reliable as those on CT images and that the measurements have good inter- and intrarater reliability. Therefore, in this study, we compared PTS measurements on lateral EOS radiographs to those on CT scans.

## Material and methods

We retrospectively searched our radiological database for patients who underwent an EOS scan and an additional rotational CT scan of their lower extremities between January and December 2019. The inclusion criterion was that both scans were obtained within 1 year. The exclusion criteria were the presence of advanced OA at the bony deformation stage and previous intra-articular fractures of the knee. Twenty-nine patients met the criteria; two patients had one knee excluded because of massive tibial bone loss due to destructive OA. Therefore, 56 knees were included in the study. The mean age of the 29 patients (18 women, 11 men) was 36 years (range = 17–63 years). This study was approved by the Swiss cantonal ethics committee. A radiologist and an orthopaedic surgeon performed the measurements at two time points with 2 months in between. Medial and lateral PTSs were determined on both the EOS radiographs and the CT scans, as described below.

### EOS measurements

**Fig. 1:**
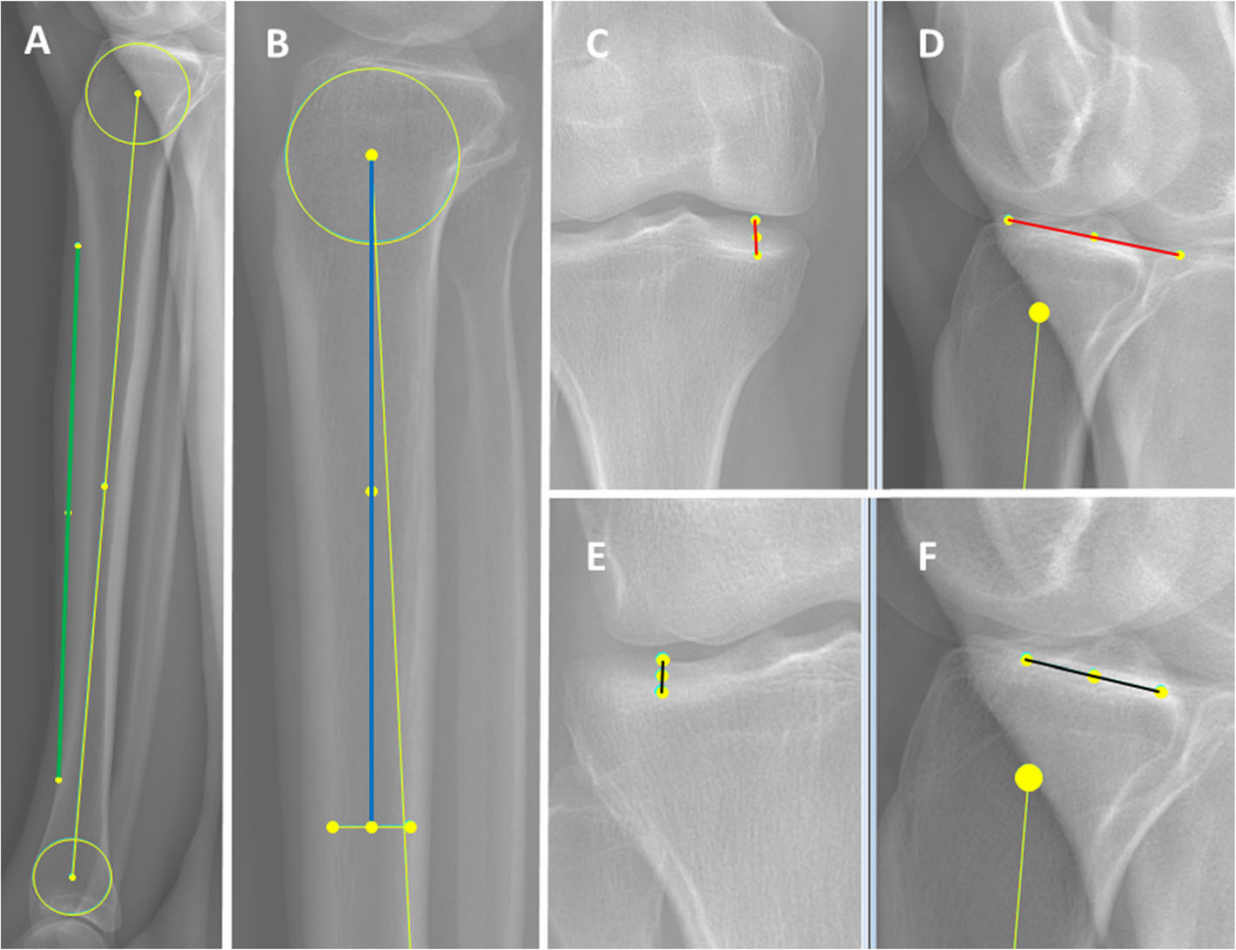
Depiction of the reference axes and slope measurements on EOS radiographs. (**A**–**F**) The yellow line is the TA on the sagittal image plane. (**A**, **B**, **D**, **F**) The green line is the ATC. (**A**) The blue line is the IMA. (**B**) The medial (**C**, **D**) and lateral (**E**, **F**) PTSs were measured on the sagittal image (**D**, **F**). The coronal image (**C**, **E**) was used mainly to identify the highest anterior point of the tibial plateau. The yellow dots are linked 3 dimensionally in the coronal and sagittal images

The EOS sterEOS® workstation 2020 with EOS software was used to measure the PTS on EOS radiographs. During EOS image acquisition, the patient stood with the right foot slightly in front of the left foot. This facilitated side identification and avoided or reduced overlay. The 3D mode of the software was used for the measurements. The physician was able to look at both 2D planes. However, setting the cursor on one plane automatically set a mark on the other plane, which allowed for 3D identification of structures. This feature made it possible to differentiate between the lateral and medial tibial compartments as well as to determine the exact location of a chosen point. All measurements were performed on the sagittal image plane, and the coronal plane was used for 3D identification. The TA was determined according to the method of Lipps et al. [16]. A circle was drawn in the proximal tibia, within the proximal, anterior, and posterior cortical borders. A second circle was drawn in the distal tibia in the same manner, touching the anterior, posterior, and distal cortices. The line between the midpoints of these circles is the TA (Fig. 1A). Two additional axes were defined. The ATC is a line at the anterior outer tibial cortex that connects a point 8 cm distal to the knee joint with a point 5 cm proximal to the ankle joint (Fig. 1A). The intramedullary axis (IMA) is a line that connects the centre of the proximal tibia (proximal circle) with the midpoint of the medullary canal 15 cm distal to the knee joint (Fig. 1B). The angles between the TA, ATC, and IMA were measured. To measure the PTS, a line connecting the highest anterior and posterior points of the lateral and medial tibial plateau was drawn (Fig. 1D, F). The points were double-checked for correctness on the coronal plane. Then, the PTS was measured using the TA as a reference.

### CT measurements

Measurements on the CT scans were performed using our standard picture archiving and communication system (PACS). Multiplanar reformation (MPR) was used to achieve 3D alignment in all three planes, standardise the initial situation, and avoid any rotational, varus/valgus, or flexion/extension malpositioning that could lead to incorrect measurements. Thus, the frontal plane was rotationally aligned with the posterior tibial cortex on the most proximal axial plane that still showed the tip of the fibula. In the coronal and sagittal planes, the longitudinal axis was adjusted so that it intersected the proximal tibia and ankle joint centrally [17]. Next, the sagittal image on which the insertion of the PCL into the tibia and the intercondylar eminence was seen was identified. Then, proximal and distal circles were drawn on the image to define the tibial axis, in the same manner as described for the EOS radiographs. The PTS was measured on the sagittal image in the same manner as described above, exactly in the centre of the medial or lateral compartment.

### Statistical analysis

IBM SPSS Statistics ver. 25 (IBM Corp., Armonk, NY, USA) was used for data analysis. The Kolmogorov-Smirnov test was used to test for a normal distribution. Because the data were normally distributed, they were reported as a mean with a 95% confidence interval (CI). Intraclass correlation (ICC) was calculated to assess inter- and intrarater reliability. Two-way mixed measures checked for consistency. ICC is presented with a 95% CI. ICC < 0.40 was rated as poor, between 0.40 and 0.59 fair, between 0.60 and 0.74 good, and between 0.75 and 1.00 excellent [18]. The Student *t* test and the Pearson correlation were used to compare the results of both imaging modalities, with *P* < 0.05 considered statistically significant. All measurements of both readers (two reads each) were included in the data analysis (*n* = 224).

## Results

The mean medial PTS was 8.5° (95% CI, 8.1–8.9°) on EOS images and 7.7° (95% CI, 7.3–8.1°) on CT images, while the lateral PTS was 7.4° (95% CI, 6.9–7.9°) on EOS images and 7.0° (95% CI, 6.5–7.4°) on CT images (Table 1). The Student *t* test showed significant differences (*P* < .001) between the medial slope measurements on the EOS and CT images, but no significant differences (*P* = .065) between the lateral slope measurements. The mean difference between the medial slope of the two modalities was 0.8° (95% CI, 0.5–1.1°) and that between the lateral slope of the two modalities was 0.4° (95% CI, 0.0–0.9°), with the higher values measured on the EOS images. The Pearson correlation (correlation coefficient *r*) showed a significant correlation between the medial (*r* = .721, *P* < .001, *n* = 224) and lateral (*r* = .498, *P* < .001, *n* = 224) PTS measurements on the EOS and CT images. The ATC reference axis yielded a mean slope that was 2.3° (95% CI, 2.2–2.4°) higher than that of the TA. The IMA yielded a mean slope that was 0.7° (95% CI, 0.5–0.8°) higher than that of TA (Table 1).

**Table 1:** Posterior medial and lateral tibial slopes and reference axes measured on CT images and EOS radiographs by two readers

	Posterior tibial slope				Differences between axes on EOS	
	EOS medial	EOS lateral	CT medial	CT lateral	TA-ATC	TA-IMA
Reader 1^a^	8.5 (7.9–9.0)	7.4 (6.8–8.1)	8.1 (7.6–8.7)	7.0 (6.4–7.7)	2.3 (2.2–2.5)	0.7 (0.5–1.0)
Reader 2^a^	8.5 (8.0–9.1)	7.4 (6.7–8.4)	7.2 (6.6–7.9)	6.9 (6.3–7.6)	2.2 (2.1–2.3)	0.6 (0.4–0.9)
Overall^b^	8.5 (8.1–8.9)	7.4 (6.9–7.9)	7.7 (7.3–8.1)	7.0 (6.5–7.4)	2.3 (2.2–2.4)	0.7 (0.5–0.8)

Values in degrees (°) are the mean (95% CI)

*TA* Tibial axis, *ATC* Anterior tibial cortex, *IMA* Intramedullary axis

^a^*n* = 112

^b^*n* = 224

The interrater reliability (ICC) with respect to the medial and lateral PTSs measured on the EOS (0.880, 0.765) and CT (0.884, 0.887) images was excellent, as well as for the measured differences between TA and ATC (0.910) and between TA and IMA (0.966). The intrarater reliability (ICC) of reader 1 with respect to the medial and lateral PTSs measured on EOS (0.927, 0.889) and CT (0.956, 0.955) images was excellent, as well as for the measured differences between TA and ATC (0.960) and between TA and IMA (0.986). Reader 2 also showed excellent intrarater reliability (Table 2).

**Table 2:** Inter- and intrarater reliability (ICC) of the PTS measurements of two readers

	Tibial slope				Differences between axes on EOS	
	EOS	EOS	CT	CT	TA-ATC	TA-IMA
	Medial	Lateral	Medial	Lateral		
Interrater	0.880 (0.826–0.917)	0.765 (0.658–0.838)	0.884 (0.832–0.920)	0.887 (0.836–0.922)	0.910 (0.869–0.938)	0.966 (0.951–0.977)
Intrarater 1	0.927 (0.876–0.957)	0.889 (0.810–0.935)	0.956 (0.925–0.974)	0.955 (0.923–0.973)	0.96 (0.932–0.977)	0.986 (0.976–0.992)
Intrarater 2	0.943 (0.903–0.967)	0.975 (0.958–0.986)	0.868 (0.774–0.922)	0.894 (0.820–0.938)	0.911 (0.848–0.948)	0.980 (0.966–0.988)

*ICC* two-way mixed measures; consistency (95% CI)

< 0.40 = poor, 0.40–0.59 = fair, 0.60–0.74 = good, 0.75–1.00 = excellent

*TA* Tibial axis, *ATC* Anterior tibial cortex, *IMA* Intramedullary axis

## Discussion

This study aimed to investigate the reliability and reproducibility of PTS measurements on EOS radiographs. We hypothesised that the PTS can be measured on EOS radiographs with good inter- and intrarater reliability compared to those of PTS measured on CT images. Our analysis of 56 EOS radiographs and their corresponding CT images confirmed this hypothesis. It showed a strong correlation between the PTS measurements from both modalities and excellent intra- and interrater agreement.

Currently, most surgeons routinely use LLRs in the AP view but only short knee radiographs in the lateral view to assess knee issues. The PTS cannot always be reliably measured on short knee radiographs; some studies have reported an overestimation of the PTS of more than 3° [7, 19]. Therefore, some authors have advocated using additional whole-tibia lateral radiographs [6, 7]. The clinical importance of a correct PTS adjustment in cruciate ligament revision surgery, corrective osteotomies, and TKA has been demonstrated; therefore, accurate planning of the sagittal alignment is key for successful procedures [1, 4, 20, 21].

Our mean PTS values of between 7.4° and 8.5° were in agreement with previously reported values [22]. The mean differences between the CT and EOS measurements of 0.8° for the medial PTS and 0.4° for the lateral PTS indicate that the EOS measurements were very accurate. In addition, the 95% CI values of ± 0.3° for the medial PTS and ± 0.5° for the lateral PTS show that the measurements were very accurate. A comparable study that investigated the difference between PTS measurements on CT images and conventional lateral LLRs reported CIs of ± 3–4° [6]. We believe that the statistically significant difference of 0.8° (95% CI, ± 0.3°) between the medial PTS values from the CT images and the EOS radiographs is not clinically relevant because the value is beyond the accuracy of surgery. The advantages of the EOS imaging system over standard AP LLRs are its additional lateral plane radiograph and reduction of radiation exposure for the patient by 50–80% [15]. Moreover, this system uses collimators to generate parallel beams, whereas conventional radiographs use point-source geometry, which causes spatial distortion [23]. In particular, the low radiation exposure is important for young patients who rerupture their ACL or need a corrective osteotomy.

This study has several limitations. First, it was a retrospective study that did not use a control group. However, a control group was not required for this validation study. Second, the patients were not followed up. Third, the examined radiographs showed no relevant degeneration of the knees. PTS measurements might be more difficult to obtain and not as accurate as those reported in this study if advanced OA is present. Thus, the results of this investigation and the conclusions drawn from them cannot simply be applied to TKA. Unfortunately, the data of patients with OA who underwent an EOS scan and a CT scan of a complete lower extremity were not available at our centre. Fourth, more female patients were in the study, which limits the generalisability of the results. Fifth, measuring the PTS on EOS radiographs has a learning curve and requires additional software, which certainly requires more effort than measuring the PTS using a standard PACS. Nevertheless, we think that this additional effort is worthwhile, because determining the PTS accurately with low radiation exposure for the patient is desirable when assessing knee issues, especially in young patients. In addition, the availability of the EOS system is limited, mainly because of the cost. Currently, the EOS 3D imaging system is available mostly in large centres, and some studies have reported that it is not cost-effective [12, 24].

Another limitation of this study is that the images were acquired with one foot of the patient placed slightly in front of the other to facilitate side identification. Therefore, the different positions of the legs could have affected the alignment measurements. To the best of our knowledge, no study has compared AP LLRs obtained with the patient in the abovementioned position to those obtained with the patient in a symmetric double-stance position. However, a recent study showed that flexion of the knee up to 30° does not significantly alter AP leg alignment. A combination of flexion and rotation was found to have a greater effect on the measurements. Therefore, we do not believe that the standing position used with the EOS system, which includes slight flexion without rotating one leg, will significantly change the AP leg alignment measurements [25]. Another factor that could significantly influence the alignment measurement is uneven weight-bearing on the legs [26]. Patients should be instructed to put equal weight on each leg during EOS imaging.

We believe that EOS radiographs, if available, should be the first choice for use in preoperative planning of corrective osteotomies of the lower leg and in the general assessment of the PTS. They provide the same information as conventional LLRs and the EOS system delivers less radiation, causes no spatial distortion, and provides a second plane suitable for determining the PTS of both compartments of the knee. Future studies will need to investigate whether the clinical benefits of this system justify its high cost and whether it can be reliably used to plan a TKA on patients with OA.

## Conclusion

The EOS 3D imaging system provides reliable and reproducible tibial slope measurements compared to the measurements obtained from CT images, which are the current gold standard. The EOS 3D imaging system has the advantage of lower radiation exposure for the patient, which is particularly important for young patients who require a thorough radiological evaluation of the knee. We recommend using this imaging system if possible, because it acquires more information (i.e., the additional sagittal plane) than conventional LLRs.

## Data Availability

Not applicable

## Abbreviations

ACL: Anterior cruciate ligament
AP: Anterior-posterior
ATC: Anterior tibial cortex
CI: Confidence interval
CT: Computed tomography
ICC: Intraclass correlation
IMA: Intramedullary axis
LLR: Long leg radiograph
MRP: Multiplanar reformation
OA: Osteoarthritis
PACS: Picture archiving and communication system
PCL: Posterior cruciate ligament
PTS: Posterior tibial slope
TA: Tibial axis
TKA: Total knee arthroplasty
